# Intraocular lens power calculation for silicone oil-dependent eyes

**DOI:** 10.3389/fmed.2023.1271897

**Published:** 2023-10-23

**Authors:** Leyi Wang, Xin Wang, Xuepeng Yang, Yuanyuan Si, Jiayin Wu, Yan Cui

**Affiliations:** ^1^Department of Ophthalmology, Qilu Hospital of Shandong University, Jinan, Shandong, China; ^2^Affiliated Eye Hospital of Shandong University of TCM, Jinan, Shandong, China

**Keywords:** intraocular lens power, intraocular fillers, silicone oil, theoretical formula, vitreoretinal surgery

## Abstract

**Background:**

Silicone oil tamponade is widely used in vitreoretinal surgery. In some cases, silicone oil may not be extracted for a long time or even permanently and is referred to as silicone oil-dependent eyes. In this study, we aimed to deduce a theoretical formula for calculating intraocular lens power for silicone oil-dependent eyes and compare it with clinical findings.

**Methods:**

A theoretical formula was deduced using strict geometric optical principles and the Gullstrand simplified eye model. The preoperative and postoperative refractive statuses of patients with silicone oil-dependent eyes who underwent intraocular lens implantation were studied (Group A, *n* = 13). To further test our derived theoretical formula, patients with silicone oil tamponade and first-stage intraocular lens implantation were included (Group B, *n* = 19). In total, 32 patients (32 eyes) were included in the study.

**Results:**

In group A, the calculated intraocular lens power based on our formula was 24.96 ± 3.29 diopters (D), and the actual refraction of the patients was 24.02 ± 4.14D. In group B, the theoretical intraocular lens power was 23.10 ± 3.08D, and the clinical intraocular lens power was 22.84 ± 3.42D. There was no significant difference between the theoretical and clinical refractive powers, and the intraclass correlation coefficient was 0.771 for group A and 0.811 for group B (both *p* ≤ 0.001). The mean absolute error for silicone oil-dependent eyes of the formula was 1.66 ± 2.09D. After excluding data for two patients with a flat cornea (corneal refractive power < 42D), the mean absolute error decreased to 0.83 ± 0.62D.

**Conclusion:**

A strong correlation between the theoretical and clinical intraocular lens powers was observed, and the formula we deduced can be used to calculate the intraocular lens power for silicone oil-dependent eyes. This formula will help clinicians select a more appropriate intraocular lens for patients with silicone oil-dependent eyes, especially when the corneal refractive power is ≥42D.

## 1. Introduction

Silicone oil (SO) is one of the most important intraocular fillers used in vitreoretinal surgery (1), which is usually removed 3–6 months after tamponade (2). Retention of SO may lead to complications, including glaucoma, cataracts, band keratopathy, emulsification of SO, and possible neural toxicity (3, 4). However, there is still a distinct group of patients in real-life practice who, for various reasons, must live with long-term or even permanent SO tamponade. In such patients, retinal re-detachments, severe proliferative vitreoretinopathy (PVR), repeated vitreous hemorrhage, or continually low intraocular pressure occur after SO removal (5, 6). These are called SO-dependent eyes (7).

The intraocular lens (IOL) power calculation for silicon oil-filled eyes has been evaluated. The refractive change associated with SO tamponade in pseudophakic eyes has been studied previously (8), and the ocular biometric parameters in silicone oil-filled eyes have been investigated (9, 10). Recently, a theoretical approach to determining how specific IOL powers would change when silicone oil is used in the vitreous chamber was published (11). However, there is no available formula for calculating the IOL power in SO-dependent eyes. In this study, we aimed to derive a theoretical formula for calculating IOL power according to strict geometric optical principles and compare the calculated power with the clinically determined power.

## 2. Materials and methods

In this retrospective, single-center, observational case series, we aimed to deduce a theoretical formula for IOL calculation in SO-dependent eyes. This study adheres to the principles of the Declaration of Helsinki and was approved by the ethics committee of Qilu Hospital of Shandong University (reference number: 2019052). All patients signed an informed consent form and received no stipend.

### 2.1. Theoretical formula deduction

The Gullstrand simplified eye was used to derive the theoretical formula. In this model, the refractive system of the eye is considered a compound system consisting of two coaxially thin lenses: the cornea and a crystalline lens that focus parallel incident light on the retina. The refractive indices of air, aqueous humor, and SO are 1(n_a_), 1.336(n_ah_), and 1.403(n_SO_), respectively.

The convex lens power formula is as follows:


$$F=\frac{n}{f}\text{,}$$

where F is the refractive power of a convex lens, n is the refractive index (RI) of the substance containing the lens, and f is the focal length of the lens.

The total refractive power of two coaxial thin lenses in close proximity is calculated as follows:


$$F=F_{1}+F_{2}\text{,}$$

where F is the total refractive power and $F_{1}$ / $F_{2}$ is the refractive power of the two lenses.

For the cornea and the crystalline lens separated by a certain distance, we consider the cornea as a thin lens that has moved for a distance d in the direction of the crystalline lens in the aqueous humor to calculate the total power of the two coaxial lenses. The equivalent refractive power of the cornea after movement is given as follows:


$$F_{e}=\frac{n_{ah}*F_{c}}{n_{ah}-d*F_{c}}\text{,}$$

where F_e_ is the equivalent refractive power of the cornea after movement, $n_{ah}$ is the RI of aqueous humor, F_c_ is the original refractive power of the cornea, and d is the distance between the posterior corneal vertex and the optical plane of the IOL on the visual axis.

The ‘d’ above is also called the effective lens position (ELP) (12), calculated using the Haigis formula: (13)


d=ELP=a0+a1∗AC+a2∗AL,

where AC is the preoperative anterior chamber depth; AL is the ocular axial length; and a0, a1, and a2 are the constants for the implanted IOLs. The lens constants were optimized for each type of IOL (available at http://ocusoft.de/ulib/c1.htm, accessed on 18 July 2023). Wang-Koch adjustment of AL was performed for patients with an AL of >26 mm (14).

In SO-dependent eyes, the total refractive power of a pseudophakic eye required to focus parallel incident light on the retina is calculated as follows:


$$F_{0}=F_{e}+F_{IOL}=\frac{n_{\mathrm{SO}}}{AL-ELP}\text{,}$$

where $F_{0}$ is the total refractive power of a pseudophakic eye, $F_{e}$ is the equivalent refractive power of the cornea after moving a distance d in the direction of the crystalline lens in aqueous humor, F_IOL_ is the refractive power of implanted IOL, n_SO_ is the RI of SO, AL is the ocular axial length, and d is the distance between the posterior corneal vertex and IOL posterior surface.

Thus, the refractive power of an implanted IOL is calculated as follows:

$F_{IOL}=\frac{n_{\mathrm{SO}}}{AL-ELP}-Fe=\frac{n_{\mathrm{SO}}}{AL-ELP}-\frac{n_{ah*}F_{c}}{n_{ah}-ELP*F_{c}}$

where $n_{ah}$ is the RI of aqueous humor and F_c_ is the original refractive power of the cornea.

When substituting the constants with some variables ( $n_{\mathrm{SO}}=1.403$ , $n_{ah}=1.336$ ), we obtain the following formula (refractive power is in diopters and length is in meters):

$F_{IOL}=\frac{1.403}{AL-ELP}-\frac{1.336*F_{c}}{1.336-ELP*F_{c}}$

From this formula, we can infer that the IOL power for SO-dependent eyes theoretically depends on ocular axial length, corneal radius of curvature, the refractive power of the cornea, and the IOL constant.

### 2.2. Clinical observations

Uneventful phacoemulsification, vitrectomy, SO tamponade, and IOL implantation were performed by an experienced surgeon in all patients. The exclusion criteria were as follows: having an unstable fundus (such as macular edema, macular pucker, retinal hemorrhage, or macular hole after SO extraction), SO emulsification obstructing optometric examinations, incomplete filling of the vitreous cavity, posterior capsule rupture, corneal opacity, and incomplete clinical data. A vitrectomy was performed using a standard 3-port 23-G pars plana incision. All incisions were sutured after surgery. To calculate the IOL refractive power required for the patients to achieve emmetropia, the postoperative best spectacle correction (spherical lens) was added to the refractive power of the implanted IOL. Ocular axial length (AL), preoperative anterior chamber (AC) depth, and refractive power of the cornea (F_C_) were measured using partial coherence interferometry (IOL Master 5.5xp or IOLMater 700; Carl Zeiss Meditec, Jena, Germany). Six types of IOLs [Rayner (Rayner, Worthing, United Kingdom); Akreos Adapt (Bausch + Lomb, Laval, Canada); 868UV (USIOL, Lexington, KY); Aspira-aA (HumanOptics, Erlangen, Germany); Aaren pal (Aaren Scientific, CA, United States); Tecnis PCB00 (Johnson & Johnson Surgical Vision, Irvine, CA, United States)] were implanted, and all patients were injected with SO (Oxane® 5,700; Bausch + Lomb). Postoperative best-corrected visual acuity (BCVA) and refraction results were collected at least 1 month (1.5 months to 4.5 years) after surgery.

### 2.3. Statistic analysis

The normal distribution was assessed with the Shapiro–Wilk test, and all data conformed to the normal distribution. Then the paired *t*-test was used for comparison, and correlation analysis was adopted using the intraclass correlation coefficient (ICC). Statistical significance was set at a value of *p* of <0.05. All analyses were performed using SPSS 25 software (version 25.0; IBM, Armonk, NY).

## 3. Results

In total, 32 patients (32 eyes) who received care between January 2018 and July 2023 at the Ophthalmology Department of Qilu Hospital of Shandong University were enrolled in this study (21 male and 11 female patients). The refractive power of the implanted IOL ranged from 13 diopters (D) to 31 D.

The SO-dependent group (Group A) included 13 patients (7 male and 6 female patients), and SO cannot be removed because of poor retinal or poor general conditions, or both. Specifically, four patients were diagnosed with polypoidal choroidal vasculopathy (PCV), which characteristically presents as a sub-retinal pigment epithelium (RPE) lesion (15). RPE destruction was also observed in patients with PCV, leading to suboptimal attachment of the neurosensory epithelium and RPE (16). To prevent any further complications and instability of the neuroepithelium, massive old sub-neurosensory-retinal hemorrhage in these four patients prompted the retention of the SO. Another four patients initially underwent vitrectomy with SO tamponade for rhegmatogenous retinal detachment (RRD). Among them, three patients subsequently developed superficial detachment of the peripheral retina, and the fourth patient, who had concurrent renal failure requiring hemodialysis treatment, had a significantly reduced risk of retinal hemorrhage due to the retained SO. Three patients presented with macular holes caused by fibrovascular proliferation of proliferative diabetic retinopathy (PDR) (17). In these cases, retinal detachment at the posterior pole likely or actually occurred once SO was removed. Hence, to prevent further complications, the SO was retained or refilled. The last two patients suffered severe retinal necrosis and repeated retinal detachment due to herpes virus infection. It has been reported that 30% of the patients who undergo vitrectomy with SO tamponade due to RRD following acute retinal necrosis develop recurrent retinal detachment after SO removal (18). To maximize the preservation of the patients’ surviving retinal function and vision, the SO was not removed. In fact, SO removal was attempted for a substantial proportion of SO-dependent patients in this study, but the SO had to be refilled in the same way or in a re-operation. The clinical data of the patients are described in Table 1.

**Table 1:** Data of patients with silicone oil-dependent eyes.

Number			Axial length, mm	Average K, D	Anterior chamber depth, mm	IOL implanted, D	Actual postoperative refraction (spherical), D	Theoretical IOL power	Pre-existing conditions	Visual acuity, logMAR		
	Age range	sex								Preop uncorrected	Preop, corrected	Postop, corrected
1	50s	M	23.91	40.89	2.74	26	−7.0	25.23	PCV	1.40	0.92	0.7
2	70s	F	21.83	46.46	3.01	31	−6.50	26.06	RRD	1.50	1	0.82
3	70s	F	23.98	43.23	3.24	28	−6.25	21.7	PCV	1.40	1.22	0.92
4	70s	M	24.7	41.6	2.95	17	−1.50	21.66	RRD	1.40	1.4	0.6
5	40s	M	22.61	44.35	3.34	18	+8.0	25.99	ARN	2.00	2	0.82
6	70s	F	21.01	45.83	2.68	27.5	+3.0	30.38	RRD	1.22	0.82	0.82
7	20s	F	22.35	42.98	3.04	26	+2.50	28.70	ARN	1.80	1	0.82
8	50s	F	20.37	46.31	3.27	23.5	+4	26.43	PDR	1.60	1.1	0.9
9	60s	F	22.84	42.51	2.38	25	−0.5	26.19	RRD	1.80	1	0.8
10	50s	M	23.78	44.86	2.54	20	+1.0	19.68	PDR	2.00	1.8	1.4
11	60s	M	23.92	42.85	3.27	20.5	+3	22.47	PCV	1.20	0.8	0.5
12	80s	M	21.92	44.07	3.13	23	+4.5	28.54	PCV	1.90	1.6	1.0
13	60s	M	23.4	44.95	2.69	21	+1.5	21.45	PDR	1.90	1.6	1.0

M, male; F, female; K, keratometry; D, diopters; IOL, intraocular lens; PCV, polypoidal choroidal vasculopathy; RRD, rhegmatogenous retinal detachment; ARN, acute retinal necrosis; PDR, proliferative diabetic retinopathy; op, operation.

**Figure 1:**
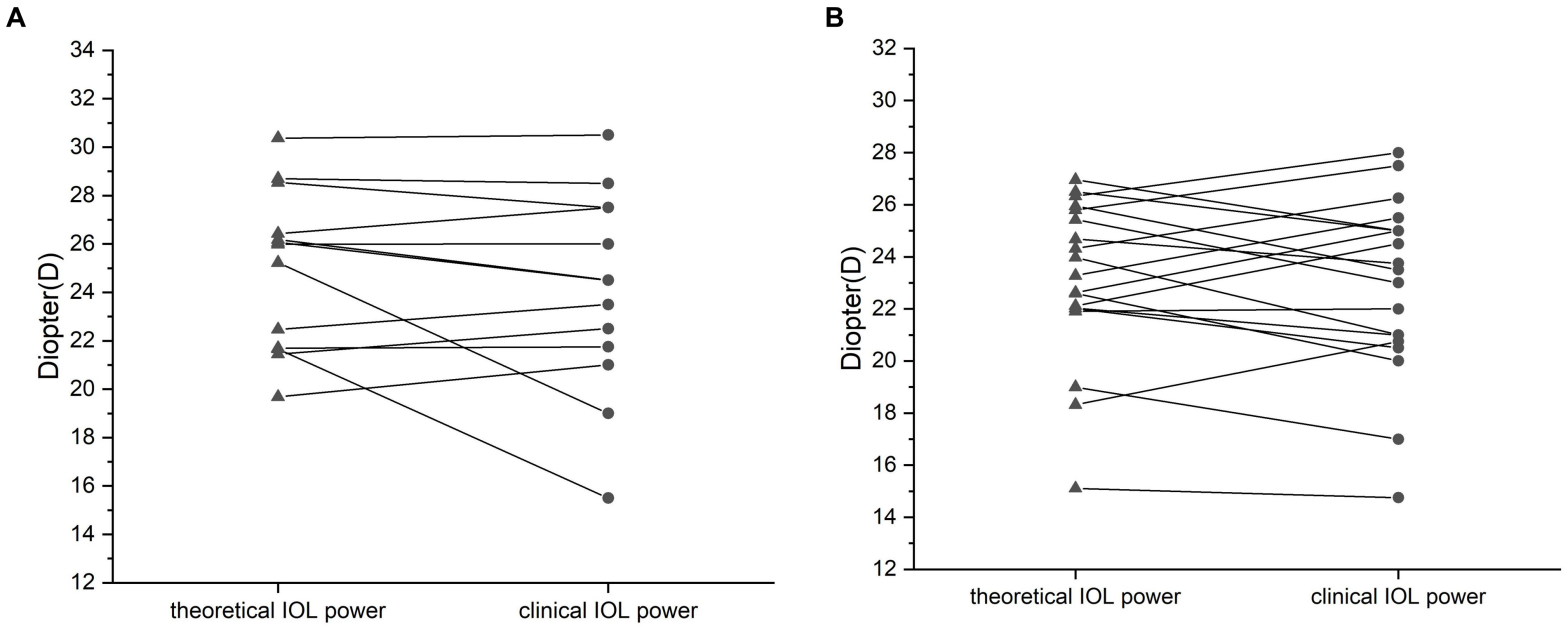
Comparison of the calculated and clinical intraocular lens (IOL) refractive powers for the silicone oil (SO)-dependent group **(A)** and the SO tamponade and first-stage IOL implantation group **(B)**. No significant difference between the theoretical and clinical refractive powers was observed. Data are expressed as the mean ± standard deviation. Group A, *p* = 0.156; group B, *p* = 0.11 (paired *t*-test).

**Figure 2:**
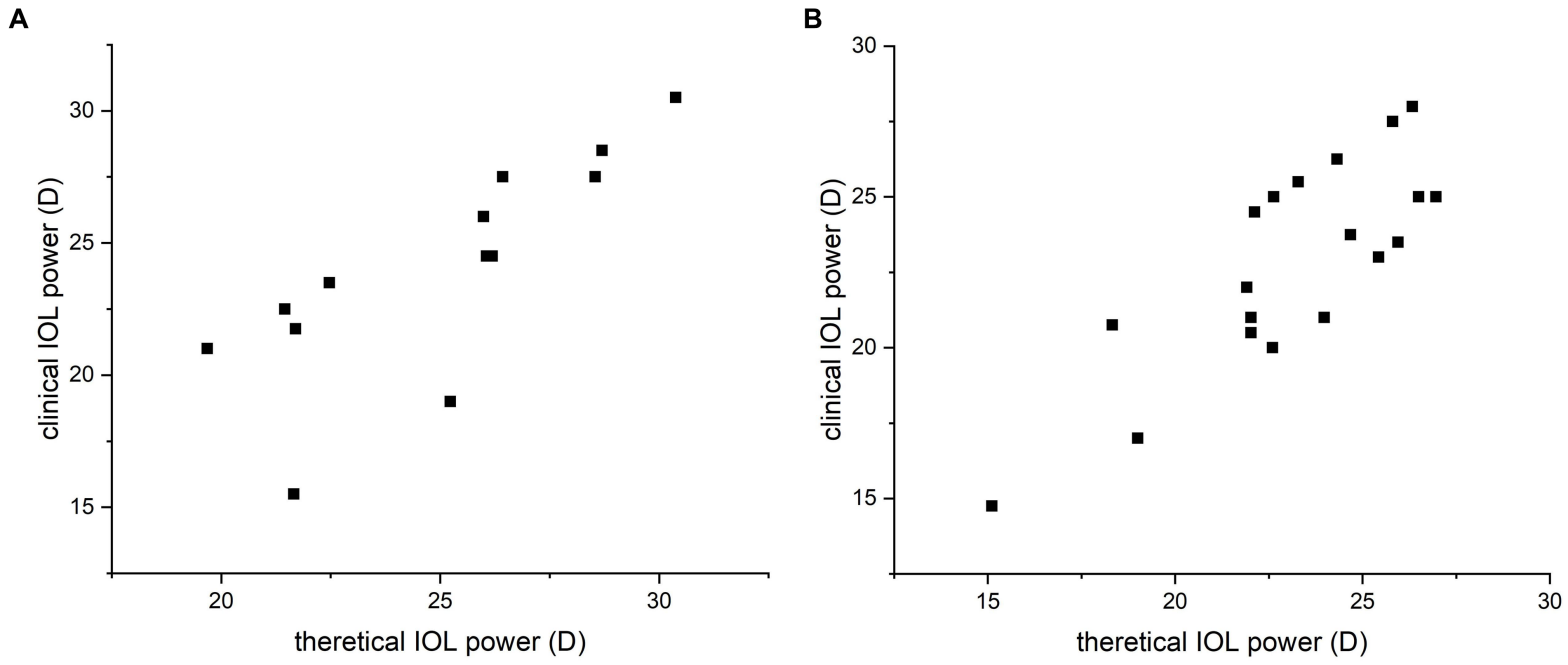
Correlation analysis of the calculated and clinical intraocular lens (IOL) refractive powers for the silicone oil (SO)-dependent group **(A)** and the SO tamponade and first-stage IOL implantation group **(B)**. Strong correlation between the theoretical and clinical refractive powers was observed by Spearman’s correlation test. Data are expressed as the mean ± standard deviation. Group A, ICC = 0.771, 95% CI: 0.405–0.924, *p* = 0.001; group B, ICC = 0.811, 95% CI: 0.573–0.923, *p* < 0.001.

In group A, the mean preoperative AC was 2.94 ± 0.31 mm, the mean F_C_ was 43.91 ± 1.76 mm, and the mean AL was 22.82 ± 1.27 mm. The theoretical IOL refractive power was 24.96 ± 3.29 D. The clinical IOL power required by the patients to achieve emmetropia was 24.02 ± 4.14 D. There was no significant difference between the theoretical and clinical IOL powers (*p* = 0.205, paired *t*-test; Figure 1A), and the theoretical and clinical IOL powers were strongly correlated (ICC = 0.771, 95% CI: 0.405–0.924, *p* = 0.001; Figure 2A). For the theoretical formula, a predicted IOL power error of <0.5 D was found in four eyes (30.77%), 1.0–1.5 D in five eyes (38.46%), 1.5–2.0 D in two eyes (15.38%), and ≥ 2.0 D in two eyes (15.38%). The mean absolute error (MAE) for the formula was 1.66 ± 2.09D. After excluding data from the two eyes with a predicted IOL power error ≥ of 2.0 D, the MAE for the formula was 0.83 ± 0.62D.

To further test our derived theoretical formula, which is designed for IOL calculation in eyes with vitreous cavities filled with SO, patients with SO tamponade and first-stage IOL implantation (implantation of the IOL during the same procedure in which vitrectomy, SO filling, and cataract extraction were performed) were included as group B. The choice of filling the vitreous cavity with SO instead of gas was made by the surgeon during the operation according to the condition of the fundus retina. The co-existence of SO and IOL in these eyes was similar to that in the SO-dependent eyes, although the application of SO in group B was eventually stopped. While the postoperative refraction data of group B for the derivation of the formula in our study were collected before the SO was removed, the actual IOL power implanted in group B was based on the recommendation of the IOL Master with the eye status of phakic; in other words, the IOL power was intended to be appropriate after the subsequent SO removal and filling of the vitreous cavity with water. The clinical data of these patients are described in Table 2. Group B included 19 patients (14 male and 5 female patients). In group B, the AC, FC, and AL were 3.03 ± 0.38 mm, 43.64 ± 1.35 mm, and 23.54 ± 1.11 mm, respectively. The theoretical IOL power was 23.10 ± 3.08 D, and the clinical IOL power was 22.84 ± 3.42 D. There was no significant difference between the theoretical and clinical IOL powers (*p* = 0.577, paired *t*-test; Figure 1B), and the theoretical and clinical IOL powers were strongly correlated (ICC = 0.811, 95% CI: 0.573–0.923, *p* < 0.001; Figure 2B).

**Table 2:** Data of patients in group B.

Number	Age	Sex	Axial length, mm	Average K, D	Anterior chamber depth, mm	IOL implanted, D	Actual postoperative refraction (spherical), D	Theoretical IOL power
1	M	70s	23.31	44.61	2.91	20.5	4	22.12
2	M	70s	24.03	42.70	3.38	21	5	22.63
3	M	60s	23.73	43.72	3.22	21	−0.5	22.02
4	M	50s	24.89	43.2	3.51	15	2	18.998
5	F	50s	21.77	45.52	2.31	25	3	26.33
6	F	60s	22.29	47.44	2.65	21.5	0.5	22.02
7	M	50s	23.15	42.32	3.76	22	3	26.5
8	F	50s	23.53	42.59	2.85	22.5	3.75	24.32
9	F	70s	23.56	43.86	3.18	20	0	22.6
10	M	50s	23	43.02	3.06	23	4.5	25.8
11	M	40s	26.53	42.2	3.45	13	1.75	15.11
12	M	60s	22.8	43.47	2.81	24	−0.5	25.95
13	M	60s	24.42	42.24	3.22	21	2.5	21.91
14	F	40s	22.29	45.74	3.15	19.5	1.25	24.68
15	M	60s	22.63	43.64	2.31	22.5	0	25.43
16	M	50s	25.03	43.09	3.27	23	1.75	18.32
17	M	50s	23.43	43.37	2.93	19	3.5	23.28
18	M	70s	23.67	43.58	2.87	22	−1	23.98
19	M	50s	23.16	42.91	2.76	22	1	26.96

M, male; F, female; K, keratometry; D, diopters; IOL, intraocular lens.

**Figure 3:**
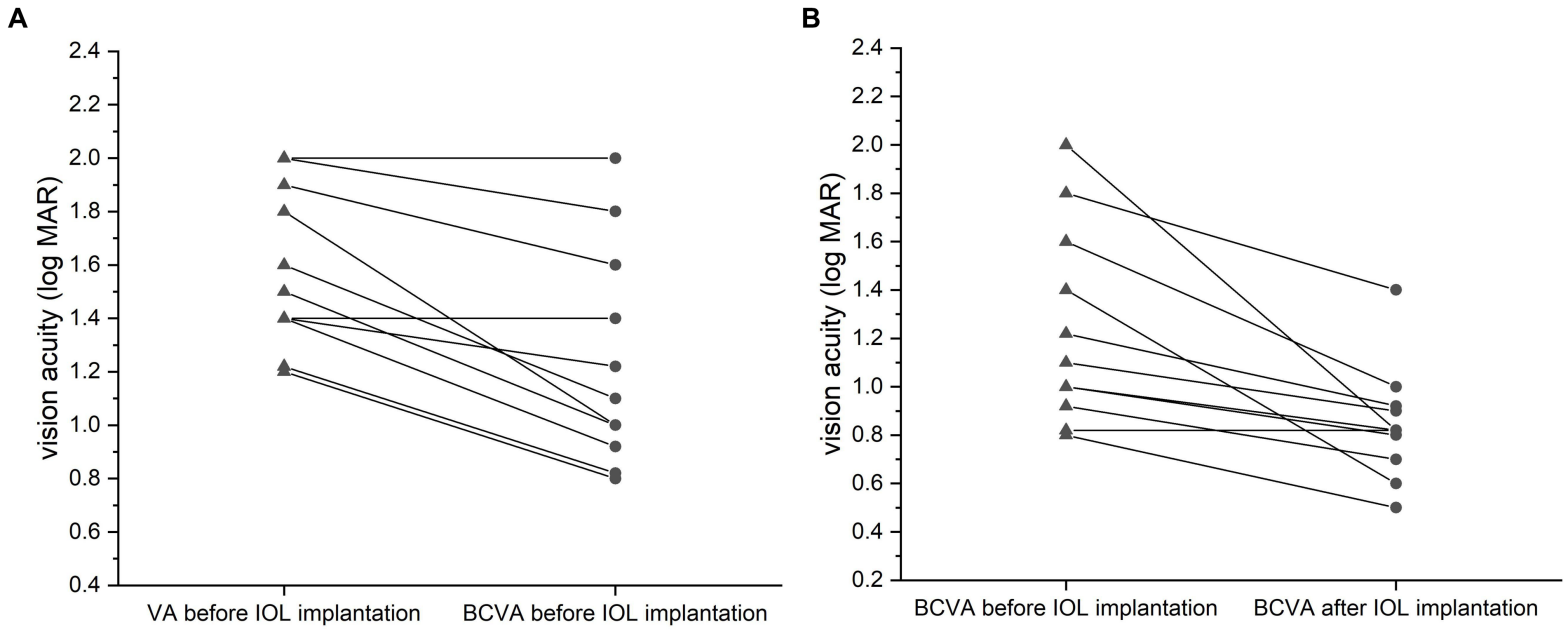
Comparison of vision acuities (VA, logMAR) for the silicone oil (SO)-dependent eyes. VA was improved after the best refractive correction before IOL implantation (**A**, *p* = 0.035, paired *t*-test). The best-corrected VA (BCVA) was further improved after the surgery relative to the preoperative BCVA (**B**, *p* = 0.043, paired *t*-test). Data are expressed as the mean ± standard deviation.

In group A, the visual acuity (VA) was improved from 1.62 ± 0.29 to 1.25 ± 0.39 logMAR after the best refractive correction before IOL implantation (*p* < 0.001, paired *t*-test; Figure 3A). After IOL implantation, the BCVA was further improved to 0.85 ± 0.22 logMAR (*p* = 0.001, paired *t*-test; Figure 3B).

## 4. Discussion

SO-dependent eyes are relatively rare in clinical practice. When SO cannot be removed for various reasons, such as poor retinal condition, poor systemic condition, or both, as in this study, but vision can be improved by spectacle correction, an IOL should be implanted to restore functional VA. The calculation method for the power of the IOLs that should be implanted in these patients has become a real challenge. Our previous attempts based on clinical experience (adding 3D–5D to the IOL power calculated by the formulas implemented in the IOL Master) have been unsuccessful, with a mean refractive error after IOL implantation of 3.79 ± 2.48 D (highest: 8 D, Table 1), creating physiological and psychological distress for both patients and doctors.

However, to our knowledge, the IOL power calculation of existing IOL power formulas, such as SRK-T (19), Haigis (13), and Barrett Universal II (20), is based on the premise that the vitreous cavity is filled with either vitreous or water. Since the refractive indices (RIs) of vitreous and water are the same (1.336), the RIs of the refractive media in contact with the anterior and posterior surfaces of the IOL, which are aqueous water and vitreous, respectively, are equal. None of the available IOL power calculation formulas provide a model for the SO-dependent eyes; even when the eye status was set to “silicone filled eye” using the formulas implemented in the IOL Master or other biometer, the calculated IOL power was intended to be appropriate after the SO had been removed and the vitreous cavity filled with water. Therefore, the IOL power for SO-dependent eyes cannot be calculated using the existing formulas. Recently, Atchison et al. applied a theoretical approach to determine how specified IOL powers should change in SO-filled eyes, but it was not clinically proven and could only be used when complete information about the IOL was released (11), which is uncommon in clinical practice. This is the first study to derive a theoretical formula for calculating the refractive power for IOL implantation in SO-dependent eyes directly using open-access IOL constants. The variables used in the formula, including AL, AC, and F_C_, can be easily obtained using the IOL Master (Carl Zeiss Meditec), ultrasonic A-scan, or corneal topography in clinics.

Numerous studies have examined the changes in biometric values of SO-filled eyes. One study found that AL measurement using optical biometry is more accurate than acoustic biometry in SO-filled eyes (9). Liu et al. reported that patients who received vitrectomy with SO tamponade for RRD repair had AL approximately 0.48 mm longer than in RRD status eyes after a mean 4.85-month duration of SO tamponade (21). In the present study, the AL data we used were obtained from the IOL Master with the status of SO-filled eyes. To address potential overestimation by the IOL Master for the AL in long eyes (22), our study applied the Wang-Koch adjustment to the AL of patients with an AL of >26 mm. Regarding anterior chamber depth (ACD), it has been reported that the increase in ACD in SO-injected patients after pars plana vitrectomy returns to preoperative values within 1 month of surgery (23).

To improve the accuracy of the calculation, we replaced the anterior chamber depth with ELP. The ELP algorithm is derived from the Haigis formula, which displays outstanding performance in vitrectomized eyes with optimized constants when predicting the accuracy of implanted IOL power (24). A strong correlation between the theoretical IOL refractive power and the clinical power for SO-dependent eyes was observed. The accuracy of the powers calculated using our formula was further confirmed in patients with SO tamponade and first-stage IOL implantation.

The IOL power calculation for SO-filled eyes has always posed a challenge for ophthalmologists (25). Even when employing optical biometry and correct calculation formulas, only a third of SO-filled eyes might achieve ±1.0 D of the target refraction after SO removal (26). Among the different types of SO-filled eyes, SO-dependent eyes present the most complex fundus conditions and structural damage. However, our study found that the predicted IOL power error was below 0.5D in 30.77% and below 2D in 84.62% of the SO-dependent eyes. These results indicate that our formula was relatively reliable and could instill confidence in ophthalmologists when selecting an IOL for a SO-dependent patient.

Interestingly, we observed that a predicted IOL power error of >2 D was found in two eyes, with both errors being extremely high: 6.23D and 6.16D, respectively (Table 1). These two patients shared a common characteristic of a flat cornea (average K < 42D). Upon excluding the data from these two patients, the MAE decreased from 1.66 ± 2.09D to 0.83 ± 0.62D. This phenomenon may be correlated with the relatively inaccurate performance of the Haigis formula in IOL calculation for eyes with flat keratometry (27). It is essential to note that the sample size of SO-dependent eyes with a flat cornea (*n* = 2) was limited, which hinders us from drawing statistically significant conclusions about the accuracy of the formula derived in this study. However, it still provides a valuable indication of the formula’s applicability, necessitating further investigations to better understand its scope of application.

When performing cataract extraction and posterior chamber IOL implantation in SO-dependent eyes, the buoyancy of SO may lead to posterior capsule elevation, additional anterior chamber instability, and an increased risk of posterior capsule rupture (28). To prevent SO leakage into the anterior chamber during surgery, pressure on the eyeball that can lead to the rupture of the suspensory ligament of the lens should be avoided. Once SO drops enter the anterior chamber, a balanced salt solution can be injected in the seated position from the superior corneal incision, and oil drops can be discharged by pressing the posterior lip of the incision.

Several potential limitations exist in this study. First, our study predicted the IOL power using data measured before IOL implantation, although some issues may arise during surgery, such as the effects of the surgical incision and changes in the position of the IOL due to viscoelastic agent residue or laxity of the capsular bag. Second, the state and amount of SO may also impact our calculation (25). However, these issues cannot be predicted; therefore, the influence of these factors is neglected in the derivation of the theoretical formula. Third, as SO-dependent eyes are relatively rare, the sample sizes and postoperative times were limited in our study. Future studies should enroll more cases involving additional postoperative time variables to further verify and improve our formula. Finally, the power of the implanted IOL in this study was calculated based on clinical experience; thus, it is unclear what the refractive error would have been if the IOL power had been calculated using our new formula. Future studies are needed to investigate the refractive error in SO-dependent eyes for which the implanted IOL power is calculated using the theoretical IOL formula proposed in this study.

## 5. Conclusion

In conclusion, we derived a theoretical formula for calculating IOL power for patients with SO-dependent eyes in this study based on geometric optical principles and the Gullstrand simplified eye model. For these severely damaged eyes, a strong correlation was found between the theoretical and clinical powers, while a predicted IOL power error of <0.5D in 30.77% and ≤ 2 D was found in 84.62% of those eyes. Thus, our formula will help clinicians select a more appropriate IOL for patients with SO-dependent eyes.

## Data availability statement

The original contributions presented in the study are included in the article/supplementary material; further inquiries can be directed to the corresponding author.

## Ethics statement

The studies involving humans were approved by the Ethics Committee of Qilu Hospital of Shandong University. The studies were conducted in accordance with the local legislation and institutional requirements. The participants provided their written informed consent to participate in this study.

## Author contributions

LW: Writing – original draft, Data curation, Formal analysis, Investigation, Methodology, Validation. XW: Data curation, Formal analysis, Investigation, Methodology, Writing – original draft, Project administration. XY: Data curation, Investigation, Methodology, Writing – original draft, Software. YS: Data curation, Investigation, Writing – original draft, Project administration. JW: Writing – original draft, Conceptualization, Funding acquisition, Methodology. YC: Conceptualization, Funding acquisition, Writing – original draft, Project administration, Supervision.

## Abbreviations

SO: Silicone oil
PVR: Proliferative vitreoretinopathy
IOL: Intraocular lens
RI: Refractive index
ELP: Effective lens position
PVC: Polypoidal choroidal vasculopathy
RPE: Retinal pigment epithelium
RRD: Rhegmatogenous retinal detachment
AL: Axial length
AC: Anterior chamber depth
FC: Refractive power of the cornea
BCVA: Best-corrected visual acuity
D: Diopters
MAE: Mean absolute error
VA: Visual acuity
PDR: Proliferative diabetic retinopathy
ACD: Anterior chamber depth
